# Cardiovascular and mortality risks following COVID-19–related versus non-COVID-19–related chronic obstructive pulmonary disease exacerbations

**DOI:** 10.1093/annalsats/aaoag084

**Published:** 2026-04-06

**Authors:** Brian K Kirui, Huiqi Li, Oskar Wallström, Lowie E G W Vanfleteren, Ailiana Santosa, Carl Bonander, Caroline Stridsman, Mats Börjesson, Fredrik Nyberg

**Affiliations:** School of Public Health and Community Medicine, Institute of Medicine, Sahlgrenska Academy, University of Gothenburg, Gothenburg, Sweden; School of Public Health and Community Medicine, Institute of Medicine, Sahlgrenska Academy, University of Gothenburg, Gothenburg, Sweden; COPD Center, Department of Respiratory Medicine and Allergology, Sahlgrenska University Hospital, Gothenburg, Sweden; Department of Internal Medicine and Clinical Nutrition, Institute of Medicine, Sahlgrenska Academy, University of Gothenburg, Gothenburg, Sweden; COPD Center, Department of Respiratory Medicine and Allergology, Sahlgrenska University Hospital, Gothenburg, Sweden; Department of Internal Medicine and Clinical Nutrition, Institute of Medicine, Sahlgrenska Academy, University of Gothenburg, Gothenburg, Sweden; Department of Respiratory Medicine, Ghent University Hospital, Ghent, Belgium; School of Public Health and Community Medicine, Institute of Medicine, Sahlgrenska Academy, University of Gothenburg, Gothenburg, Sweden; School of Public Health and Community Medicine, Institute of Medicine, Sahlgrenska Academy, University of Gothenburg, Gothenburg, Sweden; Centre for Societal Risk Research, Karlstad University, Karlstad, Sweden; Department of Public Health and Clinical Medicine/The OLIN and Sunderby Research Unit, Umeå University, Umeå, Sweden; Department of Molecular and Clinical Medicine, Institute of Medicine, Sahlgrenska Academy, University of Gothenburg, Gothenburg, Sweden; Center for Lifestyle Intervention, Department of Medicine, Geriatrics and Emergency Medicine, Sahlgrenska University Hospital, Gothenburg, Sweden; School of Public Health and Community Medicine, Institute of Medicine, Sahlgrenska Academy, University of Gothenburg, Gothenburg, Sweden

**Keywords:** COPD, exacerbations, COVID-19, target trial, register data

## Abstract

**Rationale:**

Chronic obstructive pulmonary disease (COPD) exacerbations, often triggered by viral infections like COVID-19, are associated with increased cardiovascular risk. We hypothesized that COVID-19–related exacerbations carry higher short-term cardiovascular and mortality risks than non-COVID-19–related exacerbations.

**Objectives:**

To compare 28-day risk of stroke, pulmonary embolism (PE), acute myocardial infarction (AMI), major adverse cardiovascular events (MACE), and all-cause mortality following COVID-19–related vs non-COVID-19–related COPD exacerbations and assess variation across the pandemic.

**Methods:**

Using Swedish national registers, we identified COPD with moderate (treated with oral corticosteroids with/without antibiotics) or severe (hospitalized) exacerbations from March 2020 to June 2023. Exacerbations with infection, hospitalization, or intensive care admission for COVID-19 were defined as COVID-19 related. A target trial was emulated and adjusted hazard ratios (aHRs) with 95% confidence intervals (CIs) were estimated for each outcome, stratified by exacerbation severity and COVID-19 variants.

**Results:**

Among 266 273 exacerbations (87.2% moderate, 12.8% severe), 5425 (2%) were COVID-19 related. COVID-19–related vs non-COVID-19–related moderate exacerbations were associated with risk of PE (aHR, 2.26 [95% CI, 1.49-3.42]), overall cardiovascular events (1.94 [1.47-2.56]), MACE (1.88 [1.28-2.76]), and mortality (4.58 [4.06-5.17]), but not significantly with AMI and stroke. Severe COVID-19–related exacerbations were only associated with higher mortality (1.46 [1.28-1.66]). Cardiovascular risks were highest during pre-Alpha and Delta for moderate exacerbations. Mortality remained elevated for both moderate and severe exacerbations, particularly during the same periods.

**Conclusions:**

COVID-19–related exacerbations increased MACE, short-term cardiovascular, and mortality risks, mainly for moderate exacerbations, with attenuation during Alpha and Omicron, highlighting the need for proactive cardiovascular care during respiratory outbreaks.

## Introduction

Chronic obstructive pulmonary disease (COPD) remains a leading cause of morbidity and mortality worldwide, driven by its progressive nature and the occurrence of acute exacerbations. These exacerbations accelerate lung function decline and increase the risk of hospitalization, cardiovascular events,^1^^,^^2^ and all-cause mortality.^3^^,^^4^ Systemic inflammation and related pathophysiological responses during exacerbations may contribute to increased cardiovascular risk.^5^

Respiratory infections, particularly those caused by viruses, are the most common triggers of COPD exacerbations,^6^^,^^7^ and virus-induced exacerbations tend to be more severe, often resulting in prolonged symptom duration, longer hospitalizations, and higher complication rates than nonviral exacerbations.^8^^,^^9^ Hospitalization for viral infections, including influenza, has been associated with increased cardiovascular risk,^10^ and proposed mechanisms of viral-related cardiac injury, particularly in the general population, have been described for severe acute respiratory syndrome coronavirus 2 (SARS-CoV-2) and other viral infections.^10^^,^^11^ However, not all respiratory viruses appear to exert equivalent systemic effects. Compared with common respiratory viruses such as rhinovirus, COVID-19 may induce a stronger systemic response and act as a more potent cardiovascular trigger, potentially leading to more severe exacerbations.

The sharp decline in circulation of other respiratory viruses during the pandemic,^12^^,^^13^ due to public health measures and changes in human behavior, provided a natural contrast to more clearly distinguish COVID-19–related from other COPD exacerbations, allowing more precise identification of COVID-19–specific cardiovascular risks. We hypothesized that COVID-19–related exacerbations are associated with worse short-term cardiovascular outcomes and mortality than non-COVID-19–related exacerbations. To test this, we emulated a target trial using Swedish nationwide register data to compare the 28-day risk of stroke, pulmonary embolism (PE), acute myocardial infarction (AMI), major adverse cardiovascular events (MACEs), and all-cause mortality following COVID-19 versus non-COVID-19 exacerbations of COPD, and examined whether risks varied by exacerbation severity (moderate vs severe) and across different variants of the COVID-19 pandemic.

## Methods

### Study population and data sources

We conducted a nationwide cohort study emulating a target trial, using data from the SCIFI-PEARL project (Swedish COVID-19 Investigation for Future Insights—a Population Epidemiology Approach using Register Linkage).^14^ This comprehensive initiative links individual-level information from multiple Swedish national registers covering the entire population. Demographic data were obtained from Statistics Sweden’s Register of Total Population and the Longitudinal Integrated Database for Health Insurance and Labor Market Studies (LISA).^15^ Data on emergency, inpatient, or secondary care visits were obtained from the National Patient Register (NPR),^16^ and intensive care data from the Swedish Intensive Care Register (SIR). Disease diagnoses and causes of healthcare visits were defined using the *International Classification of Diseases*, 10th Revision (ICD-10) codes. Data on dispensed medication were retrieved from the National Prescribed Drug Register (NPDR),^17^ and mortality data from the National Cause of Death Register (CDR). Additional data sources included SmiNet, the national notifiable disease database for COVID-19 testing results, and the National Vaccination Register, containing records of COVID-19 immunization. Clinical data, such as spirometry measurements, smoking status, and body mass index (BMI), were obtained from the Swedish National Airway Register (SNAR).^18^ Unique national personal identification numbers enabled individual-level linkage across registers; all data were pseudonymized prior to analysis. The study received ethical approval from the Swedish Ethical Review Authority (Dnr 2020-01800, with amendments).

## Target trial emulation

This study emulated a hypothetical target trial to estimate the effect of COVID-19–related versus non-COVID-19–related COPD exacerbations on short-term cardiovascular outcomes and all-cause mortality. The key components of the target trial protocol and their operationalization as guided by the TARGET statement^19^ using register-based data are detailed in Table E1 and illustrated in Figure 1.

**Figure 1:**
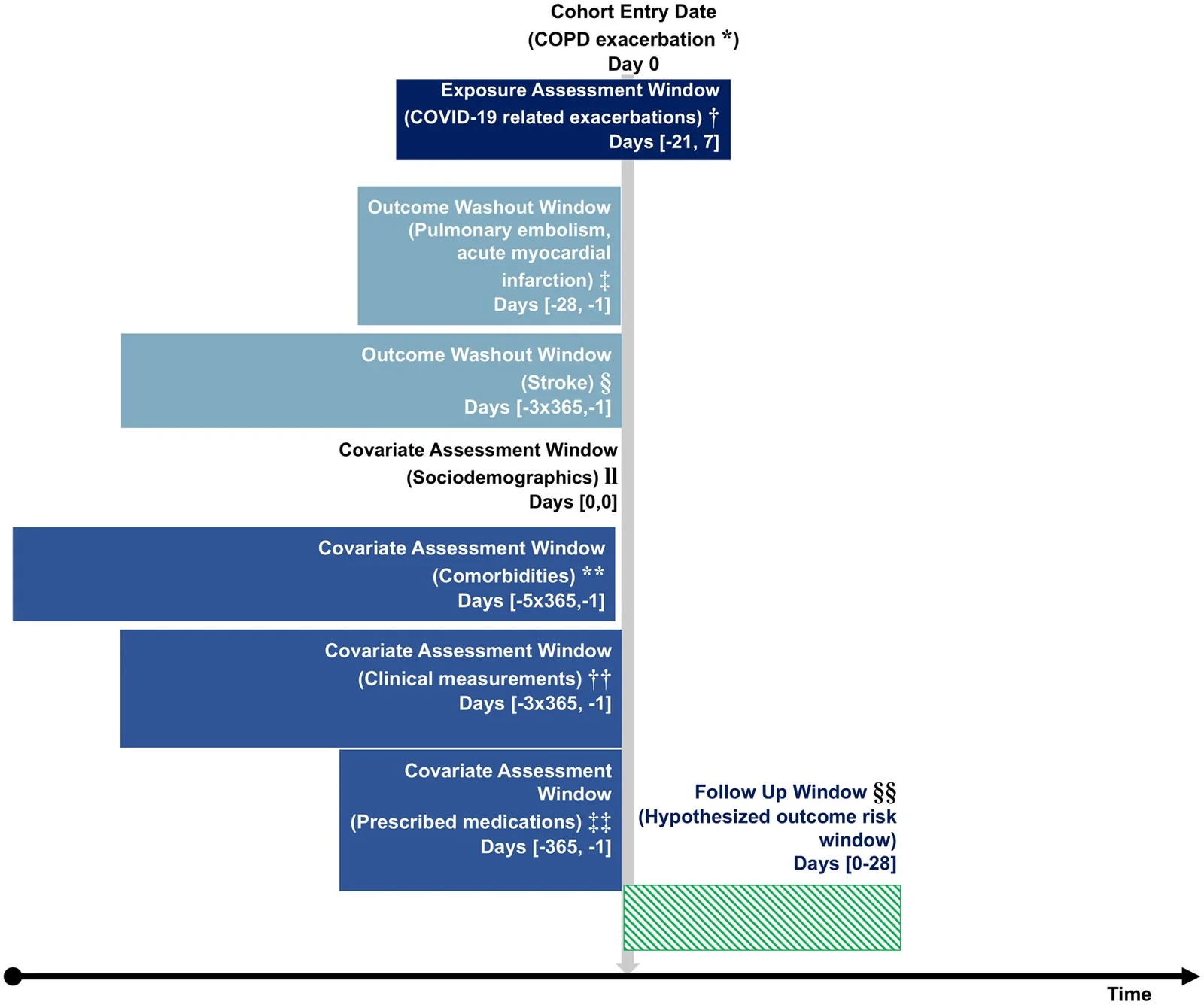
Cohort design for evaluation and follow-up of exacerbations in patients with chronic obstructive pulmonary disease (COPD) aged ≥40 years in Sweden from their exacerbation date during the study period (March 1, 2020 to June 30, 2023). ^*^COPD exacerbation date was defined by filled prescriptions for oral corticosteroids or broad-spectrum antibiotics in the National Prescribed Drugs Register (NPDR) or COPD-related hospitalizations in the National Patient Register (NPR). Exacerbations between March 1, 2020 and June 30, 2023 were included and considered distinct if ≥28 days apart from the first. ^†^Exacerbations were classified as COVID-19 related (exposed) if a positive test or medical record in primary/specialist care, hospitalization, or intensive care due to COVID-19 occurred within 21 days before to 7 days after an exacerbation; otherwise, they were classified as non-COVID-19 related. ^‡^A 28-day washout period before cohort entry ensured no prior outcome events. ^§^A 3-year washout period was applied for stroke. ^ll^Sociodemographic data from the Longitudinal Integrated Database for Health Insurance and Labor Market Studies were assessed on the cohort entry date. ^**^Comorbidities from NPR were assessed using data from the 5 years before cohort entry. ^††^The most recent clinical measurements from 3 years before cohort entry were obtained from the Swedish National Airway Register. ^‡‡^Prescribed medications from 1 year before cohort entry were retrieved from the NPDR. ^§§^A 28-day follow-up was conducted to assess cardiovascular events (acute myocardial infarction, pulmonary embolism, and stroke), a composite outcome of these events, major adverse cardiovascular events (acute myocardial infarction, stroke, or cardiovascular death), and all-cause mortality. Figure concept inspired by Schneeweiss  et al.^38^

**Table 1:** Descriptive statistics of COPD exacerbations occurring from March 1, 2020 to June 30, 2023 among patients identified in the Swedish National Patient Register and the Swedish National Airway Register, presented overall and by COVID-19–related or non-COVID-19–related status, with standardized mean differences.

Characteristic	Overall	Moderate exacerbations				Severe exacerbations			
		Total moderate	Non-COVID-19	COVID-19 related	SMD^a^	Total severe	Non-COVID-19	COVID-19 related	SMD^b^
**No.**	266 273	232 307 (87.2% of all exacerbations)	228 504	3803 (1.7% of moderate exacerbations)		33 966 (12.8% of all exacerbations)	32 344	1622 (4.8% of severe exacerbations)	
**Age, y, median [IQR]**	76.92 [70.54-82.44]	76.80 [70.34-82.34]	76.79 [70.34-82.31]	77.47 [69.76-84.00]	0.048	77.67 [71.87-83.13]	77.64 [71.84-83.10]	78.38 [72.62-83.82]	0.078
**Sex**					0.024				0.069
** Male**	103 903 (39.0%)	90 467 (38.9%)	88 943 (38.9%)	1524 (40.1%)		13 436 (39.6%)	12 742 (39.4%)	694 (42.8%)	
** Female**	162 370 (61.0%)	141 840 (61.1%)	139 561 (61.1%)	2279 (59.9%)		20 530 (60.4%)	19 602 (60.6%)	928 (57.2%)	
**Education**					0.073				0.072
** Primary**	101 880 (38.3%)	87 596 (37.7%)	86 117 (37.7%)	1479 (38.9%)		14 284 (42.1%)	13 630 (42.1%)	654 (40.3%)	
** Upper-secondary**	122 015 (45.8%)	106 925 (46.0%)	105 243 (46.1%)	1682 (44.2%)		15 090 (44.4%)	14 366 (44.4%)	724 (44.6%)	
** Tertiary**	39 179 (14.7%)	35 136 (15.1%)	34 567 (15.1%)	569 (15.0%)		4043 (11.9%)	3839 (11.9%)	204 (12.6%)	
** Unknown**	3199 (1.2%)	2650 (1.1%)	2577 (1.1%)	73 (1.9%)		549 (1.6%)	509 (1.6%)	40 (2.5%)	
**Employment**					0.119				0.062
** Unemployed**	16 118 (6.1%)	15 014 (6.5%)	14 665 (6.4%)	349 (9.2%)		1104 (3.3%)	1066 (3.3%)	38 (2.3%)	
** Retired**	35 270 (13.2%)	32 194 (13.9%)	31 755 (13.9%)	439 (11.5%)		3076 (9.1%)	2938 (9.1%)	138 (8.5%)	
** Employed**	214 885 (80.7%)	185 099 (79.7%)	182 084 (79.7%)	3015 (79.3%)		29 786 (87.7%)	28 340 (87.6%)	1446 (89.1%)	
**Comorbidity**									
** Overall CVD**	94 623 (35.5%)	81 347 (35.0%)	80 091 (35.1%)	1256 (33.0%)	0.043	13 276 (39.1%)	12 629 (39.0%)	647 (39.9%)	0.017
** Asthma**	31 271 (11.7%)	28 213 (12.1%)	27 708 (12.1%)	505 (13.3%)	0.035	3058 (9.0%)	2915 (9.0%)	143 (8.8%)	0.007
** Type 1 or 2 diabetes**	25 952 (9.7%)	22 453 (9.7%)	22 104 (9.7%)	349 (9.2%)	0.017	3499 (10.3%)	3352 (10.4%)	147 (9.1%)	0.044
** CKD**	15 510 (5.8%)	13 266 (5.7%)	13 034 (5.7%)	232 (6.1%)	0.017	2244 (6.6%)	2126 (6.6%)	118 (7.3%)	0.028
** Depression**	9315 (3.5%)	7809 (3.4%)	7646 (3.3%)	163 (4.3%)	0.049	1506 (4.4%)	1424 (4.4%)	82 (5.1%)	0.031
** Anxiety**	13 446 (5.0%)	11 028 (4.7%)	10 842 (4.7%)	186 (4.9%)	0.007	2418 (7.1%)	2305 (7.1%)	113 (7.0%)	0.006
** Cancer**	36 096 (13.6%)	32 102 (13.8%)	31 616 (13.8%)	486 (12.8%)	0.031	3994 (11.8%)	3818 (11.8%)	176 (10.9%)	0.030
**Prescribed medications**									
** ICS**	180 546 (67.8%)	156 962 (67.6%)	154 303 (67.5%)	2659 (69.9%)	0.052	23 584 (69.4%)	22 482 (69.5%)	1102 (67.9%)	0.034
** OCS**	204 788 (76.9%)	188 876 (81.3%)	186 277 (81.5%)	2599 (68.3%)	0.308	15 912 (46.8%)	15 190 (47.0%)	722 (44.5%)	0.049
** SABA**	155 407 (58.4%)	133 485 (57.5%)	131 277 (57.5%)	2208 (58.1%)	0.012	21 922 (64.5%)	20 917 (64.7%)	1005 (62.0%)	0.056
** SAMA**	39 028 (14.7%)	32 261 (13.9%)	31 661 (13.9%)	600 (15.8%)	0.054	6767 (19.9%)	6475 (20.0%)	292 (18.0%)	0.051
** LAMA**	178 236 (66.9%)	154 682 (66.6%)	152 140 (66.6%)	2542 (66.8%)	0.006	23 554 (69.3%)	22 452 (69.4%)	1102 (67.9%)	0.032
** LABA**	188 407 (70.8%)	161 905 (69.7%)	159 255 (69.7%)	2650 (69.7%)	<0.001	26 502 (78.0%)	25 283 (78.2%)	1219 (75.2%)	0.071
** LABA + LAMA combinations**	53 625 (20.1%)	45 680 (19.7%)	45 013 (19.7%)	667 (17.5%)	0.056	7945 (23.4%)	7587 (23.5%)	358 (22.1%)	0.033
** LABA + ICS combinations**	111 482 (41.9%)	98 275 (42.3%)	96 636 (42.3%)	1639 (43.1%)	0.016	13 207 (38.9%)	12 563 (38.8%)	644 (39.7%)	0.018
** Triple therapy (LABA + LAMA + ICS)**	57 395 (21.6%)	46 687 (20.1%)	45 845 (20.1%)	842 (22.1%)	0.051	10 708 (31.5%)	10 232 (31.6%)	476 (29.3%)	0.050
**No. of COVID-19 vaccinations at index**					0.288				0.342
** 0**	97 186 (36.5%)	86 323 (37.2%)	84 763 (37.1%)	1560 (41.0%)		10 863 (32.0%)	10 216 (31.6%)	647 (39.9%)	
** 1**	9621 (3.6%)	8360 (3.6%)	8222 (3.6%)	138 (3.6%)		1261 (3.7%)	1216 (3.8%)	45 (2.8%)	
** 2**	48 986 (18.4%)	42 260 (18.2%)	41 891 (18.3%)	369 (9.7%)		6726 (19.8%)	6580 (20.3%)	146 (9.0%)	
** 3**	41 735 (15.7%)	35 779 (15.4%)	34 951 (15.3%)	828 (21.8%)		5956 (17.5%)	5648 (17.5%)	308 (19.0%)	
** ≥4**	68 745 (25.8%)	59 585 (25.6%)	58 677 (25.7%)	908 (23.9%)		9160 (27.0%)	8684 (26.8%)	476 (29.3%)	

Data are No. (%) unless otherwise indicated.

Abbreviations: COPD, chronic obstructive pulmonary disease; CKD, chronic kidney disease; CVD, cardiovascular disease; ICS, inhaled corticosteroid; IQR, interquartile range; LABA, long-acting β_2_ agonist; LAMA, long-acting muscarinic antagonist; OCS, oral corticosteroid; SABA, short-acting β_2_ agonist; SAMA, short-acting muscarinic antagonist; SMD, standardized mean difference.

a SMD between COVID-19–related vs non-COVID-19–related exacerbations for moderate exacerbations.

b SMD between COVID-19–related vs non-COVID-19–related exacerbations for severe exacerbations.

### Exacerbation definitions and inclusion criteria

We included patients who experienced a COPD exacerbation between March 1, 2020 and June 30, 2023. Moderate (outpatient) exacerbations were defined using the NPDR as episodes characterized by either (1) a filled prescription for oral corticosteroids (OCS) (Anatomical Therapeutic Chemical [ATC] code H02AB); or (2) a filled prescription for respiratory-targeted antibiotics (tetracyclines [J01AA] or broad-spectrum penicillins [J01CA]) *only if* an OCS was dispensed within 28 days.

Severe (hospitalized) exacerbations were defined as hospitalizations recorded in the NPR with either (1) a primary diagnosis of COPD (ICD-10 code J44); or (2) a primary diagnosis of acute lower respiratory tract infection (J20-J22), chronic bronchitis/emphysema (J41-J43), or respiratory failure (J96), combined with a secondary COPD diagnosis (J44). These definitions represent minor modifications of previously used definitions to better align with target trial intentions.^20^^,^^21^

Distinct exacerbations were defined as events occurring at least 28 days apart, to ensure that only incident events were captured. The index date (baseline) was defined as the prescription fill date for moderate exacerbations and the hospital admission date for severe exacerbations (Figure 1).

### Exposure definition

The exposure of interest was the exacerbation being COVID-19 related. Such exacerbations were defined as those occurring in individuals with either (1) a confirmed SARS-CoV-2 polymerase chain reaction test result recorded in SmiNet; or (2) an ICD-10 diagnosis code for COVID-19 (U07.1/U07.2) listed as a primary or secondary diagnosis in the NPR or SIR within 21 days before to 7 days after the exacerbation index date (Figure 1). This time frame was chosen based on an examination of the temporal distribution of COVID-19 diagnoses relative to exacerbation onset. Exacerbations not fulfilling these criteria were classified as non-COVID-19–related and served as the comparator group.

### Outcomes and follow-up

Cardiovascular outcomes, prespecified based on their clinical relevance to COPD exacerbations, included PE (ICD-10 code I26), AMI (I21), and stroke (I61-I64). To improve statistical power, a composite cardiovascular outcome was defined as the first occurrence of any of these 3 events. To facilitate comparison with existing cardiovascular literature, a second composite outcome, MACE, was defined using established criteria and comprised AMI, stroke, or cardiovascular death (ICD-10 codes I00-I69).^22^ Outcomes were identified from the NPR from primary or secondary hospital diagnoses during hospitalizations, diagnoses, or deaths with an underlying cause of death recorded in the CDR. To enhance outcome specificity, each exacerbation had to be free from the cardiovascular outcome of interest during a washout period prior to the index date (28 days for PE and AMI; 3 years for stroke; see Table E1 footnote), as shown in Figure 1. All-cause mortality was obtained from the CDR.

Follow-up began on the index date and continued until the earliest of the following events: occurrence of the outcome of interest, emigration, death, 28 days after index date, or the end of the study period (June 30, 2023) (Figure 1). Patients who neither died nor emigrated within 28 days of an exacerbation remained eligible to reenter the target trial at subsequent exacerbations, provided that all inclusion criteria were met.

### Treatment assignment and covariate adjustment

Exposure groups were assigned based on register data as described above. To emulate conditional exchangeability, we adjusted for a predefined set of baseline covariates capturing key potential confounders of the relationship between COVID-19 and cardiovascular outcomes. These included including demographics (age, sex, education, employment) at index, comorbidities (cardiovascular disease, asthma, cancer, diabetes) recorded in the 5 years preceding index, and medication use (oral and inhaled corticosteroids, statins, COVID-19 vaccination) in the year prior to index. Additional clinical variables including spirometry results (forced expiratory volume in 1 second [FEV_1_], % predicted), BMI, and smoking status were obtained from SNAR when available, using the latest data from the 3 years before the index date. Details of all covariates and their coding are provided in Table E2.

**Table 2:** Descriptive statistics of clinical measurements and smoking habits during COPD exacerbations occurring from March 1, 2020 to June 30, 2023 among patients identified in the Swedish National Airway Register, presented overall and by COVID-19–related or non-COVID-19–related status, with standardized mean differences.

Characteristic	Overall with SNAR records	Moderate				Severe			
		Total moderate	Non-COVID-19 related	COVID-19 related	SMD^a^	Total severe	Non-COVID-19 related	COVID-19 related	SMD^b^
**No.**	147 535	130 206 (88.2% of total)	127 991	2215 (1.7% of moderate)		17 329 (11.7% of total)	16 427	902 (5.2% of severe)	
**Age, y, median [IQR]**	76.52 [70.17-81.84]	76.39 [69.97-81.73]	76.38 [69.98-81.71]	76.91 [69.00-83.29]	0.024	77.43 [71.79-82.71]	77.40 [71.77-82.68]	78.05 [72.30-83.42]	0.064
**FEV_1_ % predicted**					0.085				0.074
** ≥80**	6223 (4.2%)	5970 (4.6%)	5875 (4.6%)	95 (4.3%)		253 (1.5%)	237 (1.4%)	16 (1.8%)	
**50-79**	27 741 (18.8%)	25 921 (19.9%)	25 549 (20.0%)	372 (16.8%)		1820 (10.5%)	1735 (10.6%)	85 (9.4%)	
**30-49**	20 101 (13.6%)	17 032 (13.1%)	16 726 (13.1%)	306 (13.8%)		3069 (17.7%)	2924 (17.8%)	145 (16.1%)	
**<30**	6522 (4.4%)	5057 (3.9%)	4966 (3.9%)	91 (4.1%)		1465 (8.5%)	1393 (8.5%)	72 (8.0%)	
**Missing**	86 948 (58.9%)	76 226 (58.5%)	74 875 (58.5%)	1351 (61.0%)		10 722 (61.9%)	10 138 (61.7%)	584 (64.7%)	
**Body mass index**					0.096				0.088
**Underweight**	5731 (3.9%)	4345 (3.3%)	4250 (3.3%)	95 (4.3%)		1386 (8.0%)	1302 (7.9%)	84 (9.3%)	
**Normal**	31 222 (21.2%)	27 069 (20.8%)	26 631 (20.8%)	438 (19.8%)		4153 (24.0%)	3928 (23.9%)	225 (24.9%)	
**Overweight**	29 179 (19.8%)	26 659 (20.5%)	26 204 (20.5%)	455 (20.5%)		2520 (14.5%)	2411 (14.7%)	109 (12.1%)	
**Obese**	23 551 (16.0%)	21 203 (16.3%)	20 783 (16.2%)	420 (19.0%)		2348 (13.5%)	2225 (13.5%)	123 (13.6%)	
**Missing**	57 852 (39.2%)	50 930 (39.1%)	50 123 (39.2%)	807 (36.4%)		6922 (39.9%)	6561 (39.9%)	361 (40.0%)	
**Smoking status**					0.105				0.073
**Never smoked**	14 455 (9.8%)	13 372 (10.3%)	13 072 (10.2%)	300 (13.5%)		1083 (6.2%)	1021 (6.2%)	62 (6.9%)	
**Formerly smoked**	49 326 (33.4%)	43 992 (33.8%)	43 274 (33.8%)	718 (32.4%)		5334 (30.8%)	5055 (30.8%)	279 (30.9%)	
**Currently smokes**	24 081 (16.3%)	20 384 (15.7%)	20 063 (15.7%)	321 (14.5%)		3697 (21.3%)	3528 (21.5%)	169 (18.7%)	
**Missing**	59 673 (40.4%)	52 458 (40.3%)	51 582 (40.3%)	876 (39.5%)		7215 (41.6%)	6823 (41.5%)	392 (43.5%)	

Data are No. (%) unless otherwise indicated.

Abbreviations: FEV_1_, forced expiratory volume in 1 second; IQR, interquartile range; SMD, standardized mean difference; SNAR, Swedish National Airway Register.

a SMD between COVID-19–related vs non-COVID-19–related exacerbations for moderate exacerbations.

b SMD between COVID-19–related vs non-COVID-19–related exacerbations for severe exacerbations.

### Statistical analysis

Baseline characteristics were summarized according to exposure type and exacerbation severity. Categorical variables are reported as counts and proportions, continuous variables as medians with interquartile ranges (IQR). Standardized mean differences (SMDs) between the groups were computed to assess covariate balance, with value <0.1 typically considered indicative of reasonable balance.^23^

Incidence rates, person-time, and event counts were calculated for each exposure group. Cox proportional hazards regression models were used to estimate adjusted hazard ratios (aHRs) and 95% confidence intervals (CIs) for each outcome, comparing COVID-19–related and non-COVID-19–related exacerbations, and a *P* value <.05 considered as statistically significant. Models were adjusted for the predefined adjustment set described above (Table E1). Analyses were conducted for overall population and separately by moderate and severe exacerbations. Stratified analyses were also performed separately for vaccinated and unvaccinated individuals at baseline.

### Sensitivity analyses

Sensitivity analyses included:

1. Additional adjustment for clinical covariates (spirometry, BMI, and smoking), with missing values coded as separate categories. To reduce missing spirometry data, pre-bronchodilator values were used when post-bronchodilator measurements were unavailable.
2. Reclassification of moderate exacerbations as severe if a severe exacerbation occurred within 28 days of a moderate one.

We used cluster-robust standard errors to account for within-individual correlation across multiple exacerbations. Secondary analyses were stratified by the dominant variant of concern periods based on the index date: pre-Alpha (before January 2021), Alpha (January to June 2021), Delta (July to December 2021), and Omicron (January 2022 onward). Analyses were conducted using SAS version 9.4 (SAS Institute, Cary, NC, USA) and R version 4.4.1 (R Foundation for Statistical Computing, Vienna, Austria).

## Results

The target trial included 266 273 moderate or severe COPD exacerbations among 64 437 patients with COPD (Table 1), (mean: 4.13 exacerbations per person; 4.50 among moderate, 1.57 among severe). The majority of exacerbations (87.2%) were moderate (Table 1). COVID-19–related exacerbations fluctuated over the study period (Figure E1), but on average were relatively uncommon, comprising approximately 2.0% of all exacerbations—1.6% of moderate and 4.8% of severe (Table 1).

Baseline characteristics of individuals experiencing COVID-19–related exacerbations (Table 1) were largely similar to those with non-COVID-19–related exacerbations. Most covariates showed acceptable balance between COVID-19 and non-COVID-19–related exacerbation groups (SMD <0.10) (Table 1). Patients with COVID-19–related exacerbations were less frequently vaccinated against COVID-19 prior to the exacerbation (Table 1). Among individuals with spirometry data, most had moderate airflow limitation (FEV_1_ % predicted: 50%-79% predicted) (Table 2). Severe and very severe airflow limitation (FEV_1_ % predicted: <50%) were more frequent in those with severe exacerbations (Table 2). BMI and smoking distributions were generally similar between COVID-19–related and non-COVID-19–related severe exacerbations, while differences were slightly more pronounced for moderate exacerbations (SMDs just above 0.1; Table 2).

In the overall analyses, COVID-19–related exacerbations were associated with increased risks of cardiovascular events and mortality (Figure 2). When stratified by exacerbation severity, cardiovascular event rates were high for all severe exacerbations (Table E3). However, for moderate exacerbations, the rates were approximately twice as high following COVID-19–related versus non-COVID-19–related moderate exacerbations, while severe exacerbations showed elevated absolute rates but less pronounced relative differences between the 2 groups (Table E3). Moderate exacerbations were associated with increased risk of PE (aHR, 2.26 [95% CI, 1.49-3.42]), MACE (1.88 [1.28-2.76]), and composite cardiovascular events (1.94 [1.47-2.56]). The association was marginally significant for stroke (1.77 [1.02-3.09]) but not for AMI (1.64 [1.00-2.70]) (Figure 2; Table E4). For severe exacerbations, cardiovascular risks and MACE did not differ significantly for COVID-19–related and non-COVID-19–related exacerbations. However, all-cause mortality was significantly higher for both moderate and severe COVID-19–related exacerbations (Figure 2).

**Figure 2:**
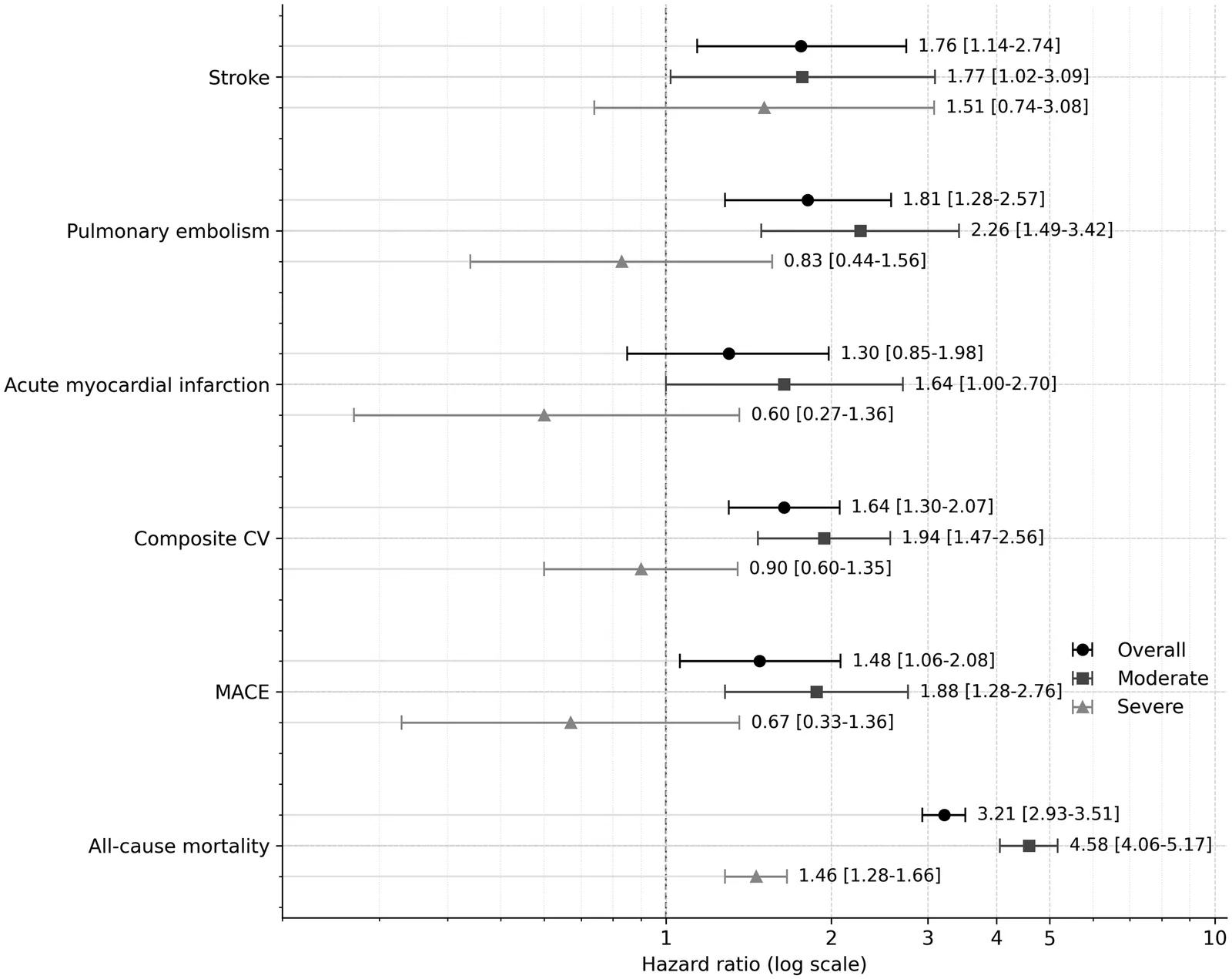
Adjusted hazard ratios and 95% confidence intervals for cardiovascular outcomes or death in COVID-19–related versus other exacerbations overall and stratified by exacerbation severity, from March 2020 to June 2023 in Sweden. Composite CV: the earliest of stroke, pulmonary embolism, or acute myocardial infarction; MACE: acute myocardial infarction, stroke, or cardiovascular death. Models adjusted for age at index, sex, education, employment, pre-index comorbidities (cardiovascular disease, asthma, cancer, diabetes), and pre-index medication use (oral corticosteroids, inhaled corticosteroids, statins) and COVID-19 vaccination. Number of events and total observations are shown in Table E6. Abbreviations: CV, cardiovascular; MACE, major adverse cardiovascular event.

**Table 3:** Risk for composite cardiovascular outcomes and all-cause mortality following COVID-19–related vs other exacerbations in Sweden (March 2020–June 2023), stratified by variant of concern and exacerbation severity.

Outcome	Variant of concern	All exacerbations	Stratified	
			Moderate exacerbations	Severe exacerbations
		aHR (95% CI)	aHR (95% CI)	aHR (95% CI)
**Composite CV**	Pre-Alpha	2.41 (1.66-3.51)	3.28 (2.16-4.98)	0.80 (0.35-1.82)
	Alpha	1.41 (0.66-3.03)	1.45 (0.59-3.53)	1.15 (0.27-4.88)
	Delta	2.60 (1.07-6.30)	3.11 (1.15-8.38)	1.43 (0.19-10.64)
	Omicron	1.19 (0.84-1.68)	1.19 (0.75-1.90)	0.89 (0.53-1.50)
**MACE**	Pre-Alpha	1.65 (0.92-2.96)	2.75 (1.53-4.94)	NA
	Alpha	0.92 (0.23-3.68)	1.23 (0.30-5.00)	NA
	Delta	2.26 (0.56-9.18)	1.53 (0.21-10.88)	3.72 (0.49-28.48)
	Omicron	1.35 (0.85-2.14)	1.51 (0.85-2.70)	0.90 (0.42-1.93)
**Mortality**	Pre-Alpha	4.48 (3.86-5.19)	6.91 (5.75-8.32)	1.61 (1.28-2.04)
	Alpha	2.11 (1.43-3.11)	3.13 (1.94-5.06)	1.10 (0.58-2.07)
	Delta	4.35 (2.95-6.41)	6.08 (3.75-9.86)	2.58 (1.37-4.83)
	Omicron	2.50 (2.19-2.85)	3.21 (2.65-3.88)	1.34 (1.13-1.60)

Number of events and total observations are shown in Table E6.

Models adjusted for age at index, sex, education, employment, pre-index comorbidities (cardiovascular disease, asthma, cancer, diabetes), and pre-index medication use (oral corticosteroids, inhaled corticosteroids, statins) and COVID-19 vaccinations.

Abbreviations: aHR, adjusted hazard ratio; CI, confidence interval; Composite CV, the earliest of stroke, pulmonary embolism, or acute myocardial infarction; CV, cardiovascular; MACE, major adverse cardiovascular event (acute myocardial infarction, stroke, or cardiovascular death); NA, models did not converge.

### Sensitivity analyses

Adjustment for additional covariates, including clinical measurements and smoking status, did not materially alter the overall findings (Table E4). When some moderate exacerbations were reclassified as severe, results remained consistent with the main findings. Hazard ratios (95% CI) for moderate exacerbations increased modestly (eg, stroke: 1.77 [1.02-3.09] vs 1.96 [1.12-3.42]; AMI: 1.64 [1.00-2.70] vs 1.80 [1.06-3.07]; mortality: 4.58 [4.06-5.17] vs 4.99 [4.40-5.65]), whereas estimates for severe exacerbations decreased slightly (eg, AMI: 0.60 [0.27-1.36] vs 0.56 [0.28-1.13]; mortality: 1.46 [1.28-1.66] vs 1.44 [1.26-1.64]). These minor shifts did not materially change the observed associations (Table E5).

In stratified analysis across SARS-CoV-2 variant periods, COVID-19–related exacerbations were associated with increased risk of the composite cardiovascular outcome in all periods, with highest estimates during the pre-Alpha and Delta periods (Table 3). For MACE, point estimates followed a similar temporal pattern but were smaller in magnitude, did not reach statistical significance, and had wide CIs. The associations were weaker, albeit not statistically significant, during Alpha and Omicron. Moderate exacerbations generally contributed most to these associations, whereas severe exacerbations showed elevated but less precise estimates. All-cause mortality risk was consistently increased for moderate exacerbations throughout the pandemic and for severe exacerbations in most variant periods, including Omicron (Table 3; Table E6). In stratified analyses by vaccination status, point estimates were generally lower for the vaccinated individuals, although precision was limited, especially for severe exacerbations due to few outcome events (Figure 3; Table E7).

**Figure 3:**
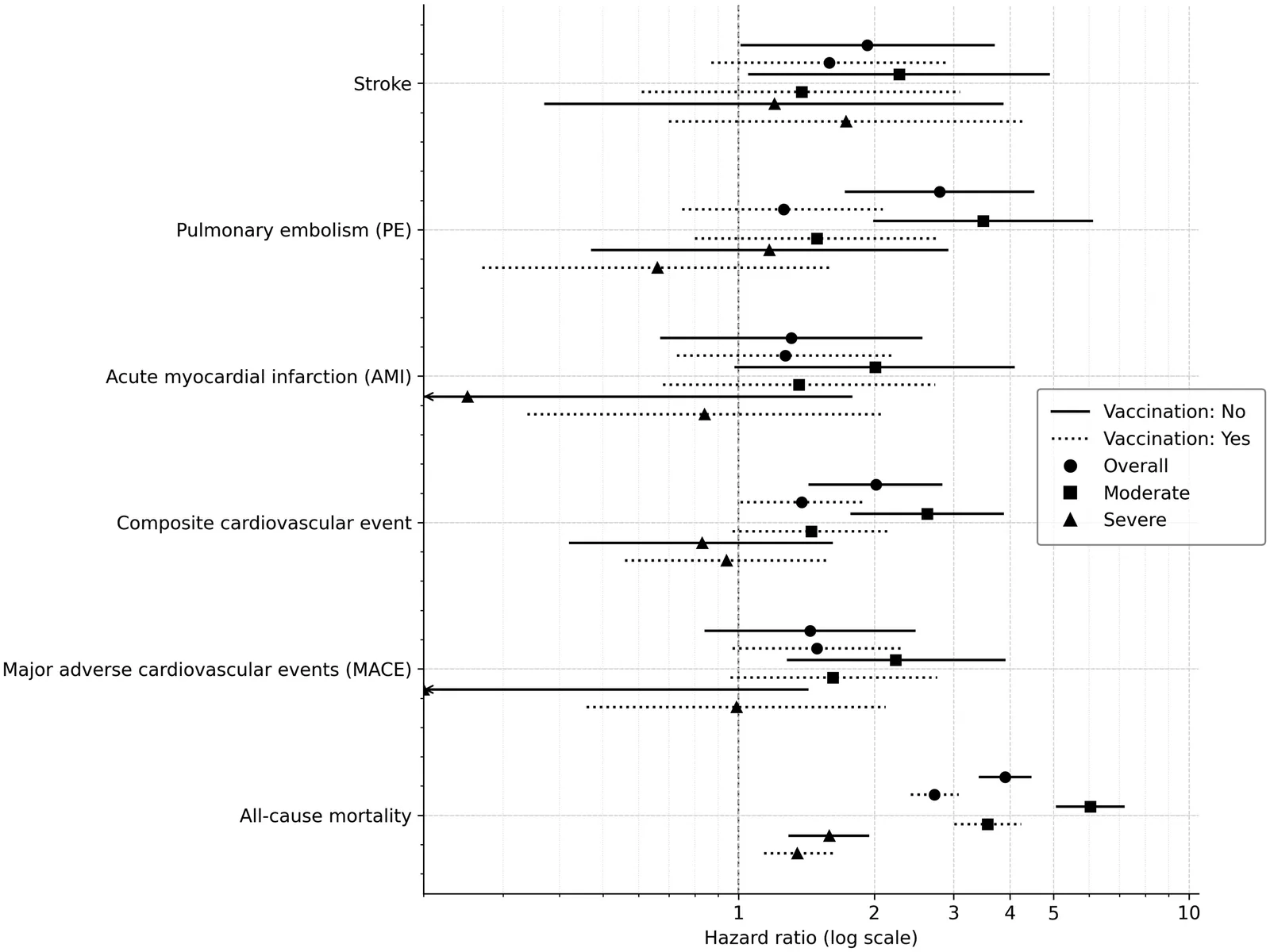
Risk for cardiovascular outcomes or death in COVID-19–related versus other exacerbations overall and stratified by exacerbation severity and baseline COVID-19 vaccination status, from March 2020 to June 2023 in Sweden. Composite CV: the earliest of stroke, pulmonary embolism, or acute myocardial infarction; MACE: acute myocardial infarction, stroke, or cardiovascular death. Models adjusted for age at index, sex, education, employment, pre-index comorbidities (cardiovascular disease, asthma, cancer, diabetes), and pre-index medication use (oral corticosteroids, inhaled corticosteroids, statins). Number of events and total observations are shown in Table E7. Abbreviations: AMI, acute myocardial infarction; CV, cardiovascular; MACE, major adverse cardiovascular event; PE, pulmonary embolism.

## Discussion

In this nationwide target trial emulation study, COVID-19–related COPD exacerbations were associated with substantially higher short-term risk of cardiovascular events and all-cause mortality than non-COVID-19–related exacerbations. These excess cardiovascular and mortality risks were most pronounced after moderate (outpatient) exacerbations although mortality was higher for COVID-19–related exacerbations across both moderate and severe (hospitalized) exacerbations. Across SARS-CoV-2 variant periods, elevated cardiovascular risk persisted for moderate COVID-19–related exacerbations, with the highest estimates during the pre-Alpha and Delta periods. Mortality remained elevated for both moderate and severe exacerbations, particularly during the same periods.

Exacerbations of COPD declined significantly during the pandemic—by up to 50% compared with pre-pandemic periods.^24^^,^^25^ We observed a low prevalence of COVID-19–related exacerbations, particularly among moderate cases, likely reflecting both the overall decline in exacerbations and greater adherence to preventive behaviors in persons with COPD. The low average also reflects sharp peaks during COVID-19 waves and low levels between them. COVID-19–related exacerbations may also have been more likely to lead to hospitalization, reducing their representation among moderate cases. Alternatively, consistent with reports of substantial declines in influenza and other major viral triggers of exacerbations during the pandemic,^12^^,^^13^ non-COVID-19 triggers may have remained the predominant drivers of exacerbations during the study period.

A previous study comparing COVID-19 patients with and without chronic respiratory disease found a higher cardiovascular risk among those with a respiratory disease, which was attributed to the type and severity of the underlying respiratory condition.^26^ Our study extends this evidence by specifically examining COPD exacerbations and quantifying the additional cardiovascular risk with SARS-CoV-2 infection. Our findings suggest that SARS-CoV-2 infection confers added cardiovascular risk beyond that associated with non-COVID-19–related exacerbations. COVID-19–related exacerbations may thus carry distinct risks related to SARS-CoV-2–specific pathophysiology. The higher relative risk seen after moderate exacerbations should be interpreted cautiously, in the context of differing baseline cardiovascular risk levels. Because baseline risk is lower after moderate than after severe exacerbations, similar absolute risk increase translates to a larger relative risk increase for moderate exacerbations. Hospitalized exacerbations have a higher baseline cardiovascular risk,^27-29^ narrowing the relative differences between COVID-19–related and non-COVID-19–related events. In contrast, moderate exacerbations, with lower baseline risk, show larger relative effects from modest absolute increases. This interpretation aligns with prior findings of lower relative risk of stroke among patients with, versus without, chronic respiratory disease hospitalized for COVID-19.^26^ Notably, when individuals with prior cardiovascular disease were excluded, the same study found no association with stroke. It is also possible that differences in clinical management, which may be greater in outpatient than inpatient settings, contribute to the larger cardiovascular risk difference between moderate COVID-19 and non-COVID-19 exacerbations.

The excess cardiovascular and mortality risks associated with COVID-19–related exacerbations persisted into later pandemic phases dominated by the less severe Omicron variants, which are generally associated with milder illness.^30^ This suggests that SARS-CoV-2–related cardiovascular effects are not solely dependent on viral virulence. These findings are consistent with prior studies reporting increased cardiovascular risk in the early weeks after COVID-19 infection^31^ and sustained cardiovascular consequences following COPD exacerbations.^32^ Several biological mechanisms have been proposed to explain these associations. COPD (particularly during exacerbations) is associated with systemic inflammation and physiologic stress/hypoxemia that may increase cardiovascular risk^7^; this risk may be further intensified by SARS-CoV-2 and other respiratory viruses via acute inflammatory and prothrombotic pathways.^33^ Collectively, these findings indicate that infection-associated cardiovascular risk may persist even with less virulent respiratory pathogens, underscoring the need for ongoing cardiovascular monitoring during and after respiratory epidemics and pandemics.

### Clinical implications

Clinicians should maintain pathogen-specific vigilance and continue emphasizing exacerbation prevention, with particular attention to short-term cardiovascular monitoring after infection–related exacerbations. Proactive cardiovascular risk management may be warranted even in nonhospitalized cases, especially during epidemic surges when hospital capacity is limited and moderate exacerbations are managed in outpatient settings. In line with clinical guidelines, the Global Initiative for Chronic Obstructive Lung Disease (GOLD) 2025 report strongly recommends vaccination to mitigate both pulmonary and cardiovascular risks.^34^ In this target trial emulation, vaccination rates were higher among patients with non-COVID-19–related exacerbations (SMD >2.9). While causality cannot be inferred, stratified adjusted analyses by baseline vaccination status showed lower point estimates for most outcomes consistent with a potential benefit of vaccination not only against COVID-19 itself, but also against COVID-19–related exacerbations and their cardiovascular consequences. This aligns with early studies that examined the cardiovascular benefits of COVID-19 vaccination.^35^^,^^36^

### Strengths and limitations

Major strengths of this study include its large, population-based design with >3 years of follow-up, enabling robust analysis of temporal trends and COVID-19 variant–specific risks. The use of comprehensive nationwide health registers enabled near-complete capture of COVID-19–related COPD exacerbations across care settings, providing a representative view of real-world presentations; the applied definition ensured inclusion across the full population spectrum, despite some uncertainty in sensitivity for moderate cases. Importantly, the study was structured using a target trial framework, emulating a hypothetical randomized trial to strengthen causal inference. Eligibility criteria were applied uniformly to exposed and unexposed groups to minimize selection bias. By anchoring follow-up at a clearly defined time zero, the target trial emulation reduces the risk of immortal time bias, addressing a key limitation of observational research. This design also allowed a clearer view of potential confounders, helping ensure exchangeability between comparison groups; however, as with all observational data, some residual confounding cannot be entirely excluded.

Our target trial emulation estimates an intention-to-treat effect defined by initial exacerbation classification. This reflects a clinically relevant contrast and avoids bias from post-baseline conditioning. Clinically, results should be interpreted as the impact of early diagnostic classification rather than the effect of persistent COVID-19 infection over follow-up.

Focusing on short-term cardiovascular outcomes reduced potential bias from COVID-19 reinfections, evolving viral variants, and shifting clinical practices—factors that complicate longer-term evaluations. Misclassification between moderate and severe exacerbations is possible in real-world clinical settings where outpatient events may progress to hospitalization. To minimize this, severity was rigorously defined and event sequences examined; sensitivity analyses reclassifying borderline moderate cases as severe did not materially alter results, suggesting nondifferential misclassification with respect to the outcome.

Some limitations should also be acknowledged. While COVID-19 diagnoses were highly specific, sensitivity may have been reduced due to evolving testing policies (relaxed testing recommendations and increased use of self-tests), potentially leading to underascertainment of moderate COVID-19–related exacerbations in the latter year of the study period. The rarity of COVID-19–related exacerbations may have limited the precision of exposure effect estimates. This is unlikely to have substantially affected our findings, given the generally higher testing of high-risk groups such as individuals with COPD, and almost mandatory testing of hospitalized cases.^37^ The interpretation also emphasized effect estimates and uncertainty rather than statistical significance. Finally, etiologic differentiation of non-COVID-19 exacerbations was limited by the absence of microbiological data.

## Conclusion

COVID-19–related COPD exacerbations were associated with worse short-term cardiovascular outcomes than non-COVID-19 exacerbations. While the cardiovascular burden of severe COPD exacerbations is well recognized, our findings show that even moderate, outpatient-managed COVID-19–related exacerbations confer excess cardiovascular and mortality risk. These results highlight the distinct cardiovascular impact of viral-induced exacerbations and the need for vigilant postinfection surveillance. Preventive strategies—including vaccination, infection control, exacerbation prevention, and proactive cardiovascular risk management—remain essential to mitigate systemic complications and strengthen resilience against future infectious threats in individuals with COPD.

## Supplementary Material

aaoag084_Supplementary_Data

## Data Availability

The individual level data used in this study are pseudonymised and sourced from Swedish healthcare registers. Interested researchers can obtain access to the data from the appropriate Swedish public data holders subject to obtaining ethics approval for the research and adhering to all relevant legislation, processes, and data protection protocols. This article has an online data supplement, which is accessible at the Supplements tab.
