# Supplementary material for: Cardiovascular and mortality risks following COVID-19–related versus non-COVID-19–related chronic obstructive pulmonary disease exacerbations
Source: Ann Am Thorac Soc. 2026 Apr 6;23(10):1476–87. doi: 10.1093/annalsats/aaoag084 (PMC13626244; doi:10.1093/annalsats/aaoag084)
Supplement: Supplementary file 1 [file aaoag084_supplementary_data.zip › 2. COPD-COVID-19 Exacerbations and CV Risk - Supplementary Revised CLEAN.pdf]

Supplementary appendix to:

Cardiovascular and mortality risks following COVID-19-related vs. non-COVID-19 COPD exacerbations.

Brian K. Kirui, Huiqi Li, Oskar Wallström, Lowie E.G.W Vanfleteren, Ailiana Santosa, Carl Bonander, Caroline Stridsman, Mats Börjesson, Fredrik Nyberg.

Table of Contents

Table E1. **Specification and emulation of a target trial evaluating short term cardiovascular risk and mortality in COVID-19-related vs. other exacerbations in COPD patients in Sweden between March 2020-Jun 2023. ....2**

Table E2: **Covariates selected in the evaluation of the of short term cardiovascular events in a population-based cohort of COPD patients in Sweden with exacerbations from 1 March 2020 until 30 Jun 2023. ....4**

Table E3. **Outcome events, person-time, and crude incidence rates (IRs) per 1,000 person-days with 95% exact Poisson confidence intervals (CIs), overall and stratified by exacerbation severity, March 2020 - June 30, 2023 ....7**

Table E4: **Unadjusted and adjusted risk for cardiovascular outcomes and death following COVID-19-related vs. other exacerbations in Sweden, March 2020-June 2023 ....8**

Table E5. **Risk for cardiovascular outcomes or death in COVID-19-related versus other exacerbations overall and stratified by exacerbation severity, from March 2020 to June 2023 in Sweden: *sensitivity analysis reclassifying some moderate exacerbations as severe* 10**

Table E6. **Number of events and total observations for composite cardiovascular outcomes and all-cause mortality following COVID-19-related vs. other exacerbations in Sweden (March 2020-June 2023), *stratified by variant of concern and exacerbation severity* 11**

Table E7. **Risk for cardiovascular outcomes and all-cause mortality following COVID-19-related vs. other exacerbations in Sweden (March 2020-June 2023), *stratified by vaccination status at baseline and exacerbation severity* 12**

Figure E1. **Monthly variation in the proportion of COVID-19-related exacerbations among COPD patients in Sweden from March 2020 to June 2023 ....13**

***Table E1*. Specification and emulation of a target trial evaluating short term cardiovascular risk and mortality in COVID-19-related vs. other exacerbations in COPD patients in Sweden between March 2020-Jun 2023.**

Protocol element	Description of the hypothetical ideal target trial	Emulation of trial with observational data
Eligibility, inclusion and exclusion criteria	Recruitment period March 2020- Jun 2023:<br><br>1) New exacerbation<br>2) Diagnosis of COPD on or before the pandemic (1 Jan 2020)<br>3) Age ≥40 years on 1 Jan 2020.<br><br>Exclusion criteria<br><br>4) Other exacerbation in the previous 28 days.<br>5) History of the specific study outcome in the respective washout period *****.	Recruit as operationalized in study<br><br>1) Same as target trial based on data from NPR and NPDR.<br>2) Diagnosis of COPD in NPR or SNAR between 2015-2019<br>3) Same as the target trial.<br><br>Exclusion criteria<br><br>4) Same as the target trial based on NPR and NPDR.<br>5) Same as the target trial based on NPR.
Exposure strategies	- *Exposed:* COVID-19-related exacerbation (exacerbation with positive COVID-19 test)<br>- *Non-exposed:* Non-COVID-19-related exacerbation (exacerbation with a negative COVID-19 test)	- *Exposed:* COVID-19-related exacerbation (confirmed by positive PCR test, a primary or secondary diagnosis in primary care, specialist visits, hospitalization records in the NPR, or intensive care admission in the intensive care register all within 21 days before or 7 days after an exacerbation record).<br>- *Non-exposed:* Non-COVID-19-related exacerbation Other exacerbations that do not meet the criteria for COVID-19-related exacerbation.
Exposure assignment	- Patients will be randomly assigned to one of the exposure strategy groups. (This is hypothetical, not practically possible).	- Not possible to randomize.<br>- Patients will be categorized to the exposure strategy aligned with their data according to the definitions above.<br>To emulate randomization, balance between the two exposure groups on cohort entry will be checked and a predefined adjustment set of covariates used to achieve balance, using model adjustment. The following baseline covariates will be included:<br><br>1. Demographics (age, sex, education, employment status).<br>2. Comorbidities (cardiovascular disease, asthma, cancer, diabetes).<br>3. Medication use (ICS, OCS, statins, vaccination).

Protocol element	Description of the hypothetical ideal target trial	Emulation of trial with observational data
		4. In sensitivity analysis, additionally clinical measurements (FEV_1, BMI) and smoking status (only available for a subset).
Follow-up	- Follow-up starts at exacerbation date<br>- Follow-up ends at the earliest of<br>1. Date of outcome of interest<br>2. 28 days from follow-up start date<br>3. Death date<br>4. Trial withdrawal date (if patient withdraws from trial)<br>5. 30 June 2023	- Same as the target trial.<br>- Same as the target trial<br>1. Same, as operationalized in the study<br>2. Same<br>3. Same<br>4. Emigration date<br>5. Same
Outcomes	- Acute myocardial infarction (AMI).<br>- Pulmonary embolism (PE).<br>- Stroke.<br>- Composite cardiovascular (CV) event (first of these three CV events).<br>- Major adverse cardiovascular events (AMI, stroke or cardiovascular death)<br>- All-cause mortality.	- Same as the target trial.<br>As operationalized in the study
Causal estimand	Per protocol effect (effect of receiving and adhering to the exposure strategy).	- Intention to treat effect (Although only assessed in the first 7 days, an initially non-COVID-19-related exacerbation may become COVID-19-related later in follow-up)
Statistical analysis	Incidence rates of outcomes in the two groups; Hazard ratios (HR) for outcomes using the non-exposed arm as the reference category.	Same; hazard ratios are adjusted, to emulate randomization.

COVID-19, coronavirus disease 2019; COPD, chronic obstructive pulmonary disease; PCR, polymerase chain reaction; NPR, National Patient Register; SNAR, Swedish National Airwa Register; ICS, inhaled corticosteroids; OCS, oral corticosteroids; BMI, body mass index; FEV_1, forced expiratory volume in one second (percent of predicted).

* 28 days for acute myocardial infarction and pulmonary embolism, 3 years for stroke: Washout periods were defined a priori and varied by cardiovascular outcome to reduce misclassification of follow-up care as new events. Stroke often involves prolonged follow-up, rehabilitation, and repeated healthcare encounters that may be coded as stroke-related visits long after the index event; therefore, a 3-year washout period was used to improve specificity for incident stroke events. Acute myocardial infarction and pulmonary embolism are generally recorded as discrete acute events, with less frequent long-term follow-up encounters coded as repeat diagnoses; accordingly, a shorter washout period consistent with the 28-day follow-up window was applied. The 28-day follow-up period was chosen based on prior evidence demonstrating that the majority of excess cardiovascular and mortality risk following COPD exacerbations occurs within this timeframe (Hawkins et al., 2024)[1].

[1] Hawkins NM, Nordon C, Rhodes K, Talukdar M, McMullen S, Ekwaru P, et al. Heightened long-term cardiovascular risks after exacerbation of chronic obstructive pulmonary disease. Heart 2024;110:702-709.

Table E2: **Covariates selected in the evaluation of the of short term cardiovascular events in a population-based cohort of COPD patients in Sweden with exacerbations from 1 March 2020 until 30 Jun 2023.**

Covariate	Units and categories	Register source; description	Lookback window before exacerbation date (index date)
**Sociodemographic characteristics**			
Age	As a continuous variable and in 10-year categories	RTB	At index date
Sex	Male, female	RTB	At index date
Education	Primary, upper-secondary, tertiary, or unknown	LISA: primary education included pre-secondary education <9, 9, or >9 years. Upper-secondary educated included 2-year high school education, secondary education <3 years or >3 years, and tertiary education included those with post-high school education. Unknown includes children with no education information or individuals with no relevant education (e.g., immigrants).	At index date
Employment status	Employed, unemployed, or retired	LISA: The employment status variable was created using the "employment" variable and stratified by age. It classifies individuals into four categories: "Employed" includes individuals who are employed and aged 65 or younger; "Unemployed" includes individuals who are unemployed and do not have entrepreneurial income; "Retired" refers to individuals who are unemployed and aged 65 or above.	At index date
**Comorbidity**	**Units/categories**	**NPR: ICD-10 codes, any mention of condition as primary or secondary diagnosis for a hospitalisation or ≥1 visit at any time**	
Overall cardiovascular disease	Yes/No	NPR: I00-I99	5 years before index
Venous thromboembolism	Yes/No	NPR: I80-I82 and I26	5 years before index
Hypertension	Yes/No	I10	5 years before index
Coronary artery disease	Yes/No	NPR: I20 -I25	5 years before index
Peripheral artery disease	Yes/No	NPR: I702, I739	5 years before index
Heart failure	Yes/No	NPR: I50	5 years before index
Asthma	Yes/No	NPR: J45	5 years before index
Type 1 or 2 diabetes	Yes/No	NPR: E10- E14	5 years before index
Chronic kidney disease	Yes/No	NPR; N181-N184, N189	5 years before index
Depression	Yes/No	NPR: F32, F33, F31	5 years before index
Anxiety	Yes/No	NPR: F41	5 years before index
Cancer	Yes/No	NPR: C00-C97	5 years before index

Prescribed medications	Units/categories	NPDR: ATC codes. At least one filled prescription in lookback window	
Inhaled corticosteroids (ICS)	Yes/No	R03BA, R03AK, R03AL08, R03AL09	1 year before index
Oral corticosteroids (OCS)	Yes/No	H02AB	1 year before index
Short-acting β_2 agonists (SABA)	Yes/No	R03AC02, R03AC03	1 year before index
Short-acting muscarinic antagonists (SAMA)	Yes/No	R03BB01, R03AL02	1 year before index
Long-acting muscarinic antagonists (LAMA)	Yes/No	R03BB01, R03BB04, R03BB05, R03BB06, R03BB07	1 year before index
Long-acting β_2 agonists (LABA)	Yes/No	R03AC12, R03AC13, R03AC18, R03AC19	1 year before index
LABA + LAMA combinations	Yes/No	R03AL03, R03AL04, R03AL05, R03AL06, R03AL07, R03AL10	1 year before index
LABA + ICS combinations	Yes/No	R03AK06, R03AK07, R03AK08, R03AK09, R03AK10, R03AK11, R03AK12, R03AK13, R03AK14	1 year before index
Triple therapy (LABA + LAMA + ICS)	Yes/No	R03AL08, R03AL09, R03AL11, R03AL12	1 year before index
Beta Blockers	Yes/No	C07	1 year before index
Statins	Yes/No	C10AA	1 year before index
ARBs	Yes/No	C09C, C09D	1 year before index
ACEIs	Yes/No	C09A, C09B	1 year before index
Antiplatelets	Yes/No	B01AC	1 year before index
Diuretics	Yes/No	C03	1 year before index
Anticoagulants	Yes/No	B01AA, B01AF	1 year before index
**COVID-19 vaccination [a]**	**Number of vaccinations received (1,2,3, and ≥ 4)**	**National Vaccination Register from the Public Health Agency of Sweden**	**On index date**
**Clinical measurements and smoking**	**Units/categories**	**Source; Description**	
Spirometry	Categorical variable with levels;<br>- FEV_1 ≥ 80% of reference: GOLD Stage 1<br>- FEV_1 50-79% of reference: GOLD Stage 2<br>- FEV_1 30-49% of reference: GOLD Stage 3<br>- FEV_1 < 30% of reference: GOLD Stage 4	SNAR; Included Forced Expiratory Volume in one second, percent of predicted (FEV_1% predicted) and Forced Ventilatory Capacity (FVC). To minimize missing data, we used the last observed post-bronchodilator values (FVCPercentAfter and FEV1PercentAfter variables in SNAR) as the primary source. If these values were missing, we substituted them with the pre-bronchodilator values (FVCPercentBefore and FEV1PercentBefore).	Last observed value 3 years before index

Body mass index (BMI)	Continuous variable and categorized into Underweight, Normal, Overweight, Obese	SNAR; Available as a variable in the SNAR dataset that was not measured for all visits that the patients made to the healthcare facilities, and therefore, the last observed BMI value before index was used for this variable, with units in Kg/M^2. Was also categorized by grouping values into four categories according to the WHO BMI classification as "underweight" (BMI < 18.5), "normal" (BMI =18.5 to 24.0), "overweight" (BMI = 25.0 to 29.9) and "obese" (BMI above 30).	Last observed value 3 years before index
Smoking status	Never, former, current smokers and missing	SNAR; available as a variable in the SNAR dataset that was not measured for all visits that the patients made to the healthcare facilities, and therefore, the last observed smoking status before index was used for this variable. Never smokers had never been smokers; former smokers quit smoking for at least 6 months; current smokers include infrequent (non-daily smokers) and frequent smokers (1 to >20 cigarettes daily).	Last observed value 3 years before index

ACEIs, angiotensin-converting enzyme inhibitors; ARBs, angiotensin receptor blockers ; ATC, Anatomical Therapeutic Chemical; BMI, body mass index; COPD, chronic obstructive pulmonary disease; ICD-10, International Classification of Diseases, version 10; LISA, Longitudinal Integrated Database for Health Insurance and Labor Market Studies; NPDR, Swedish National Prescribed Drug Register; NPR, Swedish National Patient Register; RTB, Register of Total Population; SNAR, Swedish National Airways Register; GOLD, Global Initiative for Obstructive Lung Disease.

a. In the study population, the mRNA vaccines BNT162b2 (Pfizer-BioNTech) (77.7%) and mRNA-1273 (Moderna) (14.5%) were the predominant vaccines. ChAdOx1 nCoV-19 (AstraZeneca) (7.7%) was used only in the early phase, Ad26.COV2.S (Johnson & Johnson/Janssen) and NVX-CoV2373 (Novavax) were extremely rare and used in the latter phase.

Table E3. **Outcome events, person-time, and crude incidence rates (IRs) per 1,000 person-days with 95% exact Poisson confidence intervals (CIs), *overall and stratified by exacerbation severity, March 2020 - June 30, 2023***

**Outcome**	**Exacerbation type (exposure)**	**Overall**			**Stratified**					
					**Moderate exacerbations**			**Severe exacerbations**		
		Cases	Person-days	IR [95% CI]	Cases	Person-days	IR [95% CI]	Cases	Person-days	IR [95% CI]
**Stroke**	COVID-related	21	142,915	0.15 [0.09-0.22]	13	101,532	0.13 [0.07-0.22]	8	41,383	0.19 [0.08-0.38]
	Non-COVID-related	540	7,192,687	0.08 [0.07-0.08]	437	6,341,761	0.07 [0.06-0.08]	103	850,926	0.12 [0.10-0.15]
	Total	*561*	*7,335,602*	*0.08 [0.07-0.08]*	*450*	*6,443,293*	*0.07 [0.06-0.08]*	*111*	*892,309*	*0.12 [0.10-0.15]*
**Pulmonary embolism**	COVID-related	35	142,635	0.25 [0.17-0.34]	25	101,297	0.25 [0.16-0.36]	10	41,338	0.24 [0.12-0.44]
	Non-COVID-related	953	7,186,795	0.13 [0.12-0.14]	707	6,337,680	0.11 [0.10-0.12]	246	849,115	0.29 [0.25-0.33]
	Total	*988*	*7,329,430*	*0.13 [0.13-0.14]*	*732*	*6,438,977*	*0.11 [0.11-0.12]*	*256*	*890,453*	*0.29 [0.25-0.32]*
**Acute myocardial infarction**	COVID-related	22	142,819	0.15 [0.10-0.23]	16	101,458	0.16 [0.09-0.26]	6	41,361	0.15 [0.05-0.32]
	Non-COVID-related	798	7,189,591	0.11 [0.10-0.12]	597	6,339,688	0.09 [0.09-0.10]	201	849,903	0.24 [0.20-0.27]
	Total	*820*	*7,332,410*	*0.11 [0.10-0.12]*	*613*	*6,441,146*	*0.10 [0.09-0.10]*	*207*	*891,264*	*0.23 [0.20-0.27]*
**Composite cardiovascular event**	COVID-related	77	142,047	0.54 [0.43-0.68]	53	100,875	0.53 [0.39-0.69]	24	41,172	0.58 [0.37-0.87]
	Non-COVID-related	2241	7,171,910	0.31 [0.30-0.33]	1701	6,326,190	0.27 [0.26-0.28]	540	845,720	0.64 [0.59-0.69]
	Total	*2318*	*7,313,957*	*0.32 [0.30-0.33]*	*1754*	*6,427,065*	*0.27 [0.26-0.29]*	*564*	*886,892*	*0.64 [0.58-0.69]*
**Major adverse cardiovascular events (MACE)**	COVID-related	35	142,607	0.25 [0.17-0.34]	27	101,287	0.27 [0.18-0.39]	8	41,320	0.19 [0.08-0.38]
	Non-COVID-related	1118	7,183,929	0.16 [0.15-0.17]	881	6,335,151	0.14 [0.13-0.15]	237	848,778	0.28 [0.24-0.32]
	Total	*1153*	*7,326,536*	*0.16 [0.15-0.17]*	*908*	*6,436,438*	*0.14 [0.13-0.15]*	*245*	*890,098*	*0.28 [0.24-0.31]*
**All-cause mortality**	COVID-related	529	143164	3.70 [3.39-4.02]	299	101,709	2.94 [2.62-3.29]	230	41,455	5.55 [4.85-6.31]
	Non-COVID-related	6996	7,198,854	0.97 [0.95-0.99]	3865	6,346,690	0.61 [0.59-0.63]	3131	852,164	3.67 [3.55-3.81]
	Total	*7525*	*7,342,018*	*1.02 [1.00-1.05]*	*4164*	*6,448,399*	*0.65 [0.63-0.67]*	*3361*	*893,619*	*3.76 [3.64-3.89]*

COVID-related, confirmed by positive Polymerase Chain Reaction test, a primary or secondary diagnosis in primary care, specialist visits, hospitalization records in the National Patient Register, or intensive care admission in the Intensive Care Register all within 21 days before or 7 days after an exacerbation record.
Note, composite CV was the earliest of stroke, PE or AMI, and MACE was the earliest of stroke, AMI or cardiovascular death defined in methods.

***Table E4:* Unadjusted and adjusted risk for cardiovascular outcomes and death following COVID-19-related vs. other exacerbations in Sweden, March 2020-June 2023**

**Outcome**	**Model**	**Overall (266273 observations)**		**Stratified: Moderate exacerbations (232307 observations)**		**Stratified: Severe exacerbations (33966 observations)**	
		**HR [95%CI]**	**N events**	**HR [95%CI]**	**N events**	**HR [95%CI]**	**N events**
**Stroke**	Model 1: unadjusted	1.96 [1.26-3.03]	561	1.86 [1.07-3.23]	450	1.60 [0.78-3.28]	111
	Model 2: age-sex adjusted	1.92 [1.24-2.97]	561	1.84 [1.06-3.19]	450	1.56 [0.76-3.20]	111
	Model 3: model 2 + comorbidities and medications	1.77 [1.14-2.74]	561	1.77 [1.02-3.09]	450	1.54 [0.75-3.14]	111
	Model 4: model 3 + vaccination ***(main model)****	*1.76 [1.14-2.74]*	*561*	*1.77 [1.02-3.09]*	*450*	*1.51 [0.74-3.08]*	*111*
	Model 5: model 4 + smoking	1.76 [1.14-2.73]	561	1.77 [1.02-3.08]	450	1.52 [0.74-3.11]	111
	Model 6: model 4 + BMI	1.78 [1.15-2.76]	561	1.79 [1.03-3.12]	450	1.51 [0.74-3.11]	111
	Model 7: model 4 + FEV_1	1.75 [1.13-2.72]	561	1.76 [1.01-3.07]	450	1.50 [0.74-3.07]	111
	Model 8: model 4 + BMI + smoking + FEV1	1.76 [1.13-2.73]	561	1.76 [1.01-3.07]	450	1.52 [0.74-3.11]	111
**Pulmonary embolism**	Model 1: unadjusted	1.85 [1.31-2.62]	988	2.21 [1.46-3.35]	732	0.83 [0.44-1.57]	256
	Model 2: age-sex adjusted	1.85 [1.31-2.62]	988	2.22 [1.46-3.36]	732	0.83 [0.44-1.57]	256
	Model 3: model 2 + comorbidities and medications	1.81 [1.28-2.57]	988	2.26 [1.49-3.42]	732	0.83 [0.44-1.56]	256
	Model 4: model 3 + vaccination ***(main model)****	*1.81 [1.28-2.57]*	*988*	*2.26 [1.49-3.42]*	*732*	*0.83 [0.44-1.56]*	*256*
	Model 5: model 4 + smoking	1.81 [1.28-2.57]	988	2.26 [1.50-3.43]	732	0.83 [0.44-1.56]	256
	Model 6: model 4 + BMI	1.80 [1.27-2.56]	988	2.25 [1.49-3.40]	732	0.83 [0.44-1.55]	256
	Model 7: model 4 + FEV_1	1.81 [1.28-2.56]	988	2.26 [1.49-3.42]	732	0.83 [0.44-1.56]	256
	Model 8: model 4 + BMI + smoking + FEV_1	1.80 [1.27-2.54]	988	2.25 [1.49-3.41]	732	0.82 [0.44-1.55]	256
**Acute myocardial infarction**	Model 1: unadjusted	1.38 [0.91-2.11]	820	1.67 [1.02-2.75]	613	0.61 [0.27-1.38]	207
	Model 2: age-sex adjusted	1.35 [0.88-2.06]	820	1.63 [0.99-2.68]	613	0.61 [0.27-1.37]	207
	Model 3: model 2 + comorbidities and medications	1.30 [0.85-1.99]	820	1.64 [1.00-2.70]	613	0.60 [0.27-1.36]	207
	Model 4: model 3 + vaccination ***(main model)****	*1.30 [0.85-1.98]*	*820*	*1.64 [1.00-2.70]*	*613*	*0.60 [0.27-1.36]*	*207*
	Model 5: model 4 + smoking	1.30 [0.85-1.99]	820	1.65 [1.00-2.71]	613	0.60 [0.27-1.35]	207
	Model 6: model 4 + BMI	1.29 [0.85-1.98]	820	1.64 [1.00-2.70]	613	0.60 [0.26-1.34]	207
	Model 7: model 4 + FEV_1	1.29 [0.84-1.97]	820	1.63 [0.99-2.68]	613	0.60 [0.27-1.35]	207
	Model 8: model 4 + BMI + smoking + FEV_1	1.29 [0.84-1.97]	820	1.64 [1.00-2.70]	613	0.60 [0.26-1.35]	207

**Composite cardiovascular event**	Model 1: unadjusted	1.73 [1.38-2.18]	2318	1.95 [1.48-2.58]	1754	0.91 [0.61-1.37]	564
	Model 2: age-sex adjusted	1.71 [1.36-2.16]	2318	1.94 [1.47-2.56]	1754	0.90 [0.60-1.36]	564
	Model 3: model 2 + comorbidities and medications	1.64 [1.30-2.07]	2318	1.94 [1.47-2.56]	1754	0.90 [0.60-1.35]	564
	Model 4: model 3 + vaccination ***(main model)****	*1.64 [1.30-2.07]*	*2318*	*1.94 [1.47-2.56]*	*1754*	*0.90 [0.60-1.35]*	*564*
	Model 5: model 4 + smoking	1.64 [1.30-2.07]	2318	1.94 [1.47-2.57]	1754	0.90 [0.60-1.35]	564
	Model 6: model 4 + BMI	1.64 [1.30-2.06]	2318	1.94 [1.47-2.56]	1754	0.89 [0.59-1.34]	564
	Model 7: model 4 + FEV_1	1.63 [1.30-2.06]	2318	1.93 [1.46-2.55]	1754	0.89 [0.59-1.35]	564
	Model 8: model 4 + BMI + smoking + FEV_1	1.63 [1.29-2.05]	2318	1.93 [1.46-2.56]	1754	0.89 [0.59-1.34]	564
**Major adverse cardiovascular events (MACE)**	Model 1: unadjusted	1.58 [1.13-2.21]	1153	1.92 [1.31-2.81]	908	0.69 [0.34-1.40]	245
	Model 2: age-sex adjusted	1.55 [1.11-2.17]	1153	1.89 [1.29-2.77]	908	0.69 [0.34-1.39]	245
	Model 3: model 2 + comorbidities and medications	1.48 [1.06-2.08]	1153	1.88 [1.28-2.76]	908	0.68 [0.34-1.37]	245
	Model 4: model 3 + vaccination ***(main model)****	*1.48 [1.06-2.08]*	*1153*	*1.88 [1.28-2.76]*	*908*	*0.67 [0.33-1.36]*	*245*
	Model 5: model 4 + smoking	1.48 [1.06-2.08]	1153	1.89 [1.29-2.77]	908	0.67 [0.33-1.36]	245
	Model 6: model 4 + BMI	1.48 [1.06-2.08]	1153	1.89 [1.29-2.77]	908	0.67 [0.33-1.35]	245
	Model 7: model 4 + FEV_1	1.47 [1.05-2.07]	1153	1.88 [1.28-2.75]	908	0.67 [0.33-1.36]	245
	Model 8: model 4 + BMI + smoking + FEV_1	1.47 [1.05-2.07]	1153	1.88 [1.28-2.76]	908	0.67 [0.33-1.36]	245
**All-cause mortality**	Model 1: unadjusted	3.79 [3.47-4.15]	7525	4.84 [4.29-5.45]	4164	1.50 [1.31-1.71]	3361
	Model 2: age-sex adjusted	3.61 [3.30-3.95]	7525	4.59 [4.07-5.18]	4164	1.47 [1.28-1.67]	3361
	Model 3: model 2 + comorbidities and medications	3.21 [2.93-3.51]	7525	4.58 [4.06-5.17]	4164	1.46 [1.28-1.67]	3361
	Model 4: model 3 + vaccination ***(main model)****	*3.21 [2.93-3.51]*	*7525*	*4.58 [4.06-5.17]*	*4164*	*1.46 [1.28-1.66]*	*3361*
	Model 5: model 4 + smoking	3.21 [2.93-3.52]	7525	4.59 [4.07-5.19]	4164	1.46 [1.28-1.67]	3361
	Model 6: model 4 + BMI	3.18 [2.90-3.48]	7525	4.59 [4.06-5.18]	4164	1.45 [1.27-1.66]	3361
	Model 7: model 4 + FEV_1	3.15 [2.87-3.45]	7525	4.52 [4.00-5.10]	4164	1.46 [1.28-1.67]	3361
	Model 8: model 4 + BMI + smoking + FEV_1	3.13 [2.85-3.43]	7525	4.53 [4.01-5.12]	4164	1.45 [1.27-1.65]	3361

HRs, hazard ratios; 95%CI, 95% confidence intervals; BMI, body mass index; FEV_1, forced expiratory volume in one second (percent of predicted).
* Model 4 was the main model, adjusted for age at index, sex, education, employment, pre-index comorbidities (cardiovascular disease, asthma, cancer, diabetes) and pre-index medication use (oral corticosteroids, inhaled corticosteroids, statins) and COVID-19 vaccination.

Note, composite CV was the earliest of stroke, PE or AMI, and MACE was the earliest of stroke, AMI or cardiovascular death defined in methods.

Table E5. **Risk for cardiovascular outcomes or death in COVID-19-related versus other exacerbations overall and stratified by exacerbation severity, from March 2020 to June 2023 in Sweden:** ***sensitivity analysis reclassifying some moderate exacerbations as severe***

Exacerbation severity	Outcome	Events			N			Effect *	
		Before reclassification	After reclassification	Change	N before reclassification	N after reclassification	Change	HR [95% CI] before reclassification	HR [95% CI] after reclassification
Moderate	Stroke	450	420	30	232307	226257	6050	1.77 [1.02-3.09]	1.96 [1.12-3.42]
	PE	732	653	79	232307	226257	6050	2.26 [1.49-3.42]	2.20 [1.42-3.40]
	AMI	613	507	106	232307	226257	6050	1.64 [1.00-2.70]	1.80 [1.06-3.07]
	Composite CV	1754	1546	208	232307	226257	6050	1.94 [1.47-2.56]	2.02 [1.51-2.70]
	MACE	908	781	127	232307	226257	6050	1.88 [1.28-2.76]	2.10 [1.41-3.13]
	Mortality	4164	3791	373	232307	226257	6050	4.58 [4.06-5.17]	4.99 [4.40-5.65]
Severe	Stroke	111	141	-30	33966	40016	-6050	1.51 [0.74-3.08]	1.22 [0.60-2.49]
	PE	256	335	-79	33966	40016	-6050	0.83 [0.44-1.56]	0.93 [0.52-1.64]
	AMI	207	313	-106	33966	40016	-6050	0.60 [0.27-1.36]	0.56 [0.28-1.13]
	Composite CV	564	772	-208	33966	40016	-6050	0.90 [0.60-1.35]	0.85 [0.59-1.24]
	MACE	245	372	-127	33966	40016	-6050	0.67 [0.33-1.36]	0.58 [0.31-1.09]
	Mortality	3361	3734	-373	33966	40016	-6050	1.46 [1.28-1.66]	1.44 [1.26-1.64]

HRs, hazard ratios; 95%CI, 95% confidence intervals; PE, pulmonary embolism; AMI, acute myocardial infarction; CV, cardiovascular event; MACE, major adverse cardiovascular events

* Estimates from main model presented, adjusted for age at index, sex, education, employment, pre-index comorbidities (cardiovascular disease, asthma, cancer, diabetes) and pre-index medication use (oral corticosteroids, inhaled corticosteroids, statins) and COVID-19 vaccination.

Note, composite CV was the earliest of stroke, PE or AMI, and MACE was the earliest of stroke, AMI or cardiovascular death defined in methods.

Table E6. **Number of events and total observations for composite cardiovascular outcomes and all-cause mortality following COVID-19-related vs. other exacerbations in Sweden (March 2020-June 2023),** ***stratified by variant of concern and exacerbation severity***

**Outcome**	**Variant of concern**	**Overall**		**Stratified**			
				**Moderate exacerbations**		**Severe exacerbations**	
		**N events**	**Total observations**	**N events**	**Total observations**	**N events**	**Total observations**
**Composite cardiovascular event**	Pre-Alpha	651	78,936	526	70,904	125	8,032
	Alpha	301	32,787	230	28,766	71	4,021
	Delta	389	40,351	285	35,016	104	5,335
	Omicron	977	114,199	713	97,621	264	16,578
**Major adverse cardiovascular events (MACE)**	Pre-Alpha	341	78,936	283	70,904	58	8,032
	Alpha	134	32,787	109	28,766	25	4,021
	Delta	187	40,351	145	35,016	42	5,335
	Omicron	491	114,199	371	97,621	120	16,578
**All-cause mortality**	Pre-Alpha	2,212	78,936	1,349	70,904	863	8,032
	Alpha	881	32,787	496	28,766	385	4,021
	Delta	1,183	40,351	651	35,016	532	5,335
	Omicron	3,249	114,199	1,668	97,621	1,581	16,578

HRs, hazard ratios; 95%CI, 95% confidence intervals; Composite cardiovascular event, the earliest of stroke, pulmonary embolism or acute myocardial infarction; cardiovascular; MACE, major adverse cardiovascular event: acute myocardial infarction, stroke, or cardiovascular death.
NA, models did not converge.

Models adjusted for age at index, sex, education, employment, pre-index comorbidities (cardiovascular disease, asthma, cancer, diabetes) and pre-index medication use (oral corticosteroids, inhaled corticosteroids, statins) and vaccination.

Table E7. **Risk for cardiovascular outcomes and all-cause mortality following COVID-19-related vs. other exacerbations in Sweden (March 2020-June 2023),** ***stratified by vaccination status at baseline and exacerbation severity***

Outcome	Vaccination status at baseline	Overall		Stratified			
				Moderate exacerbations		Severe exacerbations	
		HR [95%CI] [a]	N events	HR [95%CI] [a]	N events	HR [95%CI] [a]	N events
**Stroke**	No [b]	1.93 [1.01-3.70]	208	2.27 [1.05-4.91]	167	1.20 [0.37-3.87]	41
	Yes [c]	1.59 [0.87-2.91]	353	1.38 [0.61-3.10]	283	1.73 [0.70-4.28]	70
**Pulmonary embolism (PE)**	No [b]	2.79 [1.72-4.53]	350	3.49 [1.99-6.12]	274	1.17 [0.47-2.92]	76
	Yes [c]	1.26 [0.75-2.09]	638	1.49 [0.80-2.78]	458	0.66 [0.27-1.60]	180
**Acute myocardial Infarction (AMI)**	No [b]	1.31 [0.67-2.56]	299	2.01 [0.98-4.10]	232	0.25 [0.03-1.79]	67
	Yes [c]	1.27 [0.73-2.21]	521	1.36 [0.68-2.73]	381	0.84 [0.34-2.07]	140
**Composite cardiovascular event**	No [b]	2.02 [1.43-2.83]	840	2.62 [1.77-3.88]	656	0.83 [0.42-1.62]	184
	Yes [c]	1.38 [1.01-1.90]	1478	1.45 [0.97-2.18]	1098	0.94 [0.56-1.58]	380
**Major adverse cardiovascular events (MACE)**	No [b]	1.44 [0.84-2.47]	419	2.23 [1.28-3.91]	340	0.20 [0.03-1.43]	79
	Yes [c]	1.49 [0.97-2.30]	734	1.62 [0.96-2.76]	568	0.99 [0.46-2.12]	166
**All-cause Mortality**	No [b]	3.90 [3.41-4.47]	2711	6.03 [5.06-7.19]	1583	1.59 [1.29-1.95]	1128
	Yes [c]	2.72 [2.41-3.08]	4814	3.57 [3.01-4.24]	2581	1.35 [1.14-1.62]	2233

COPD, chronic obstructive pulmonary disease
Note, composite cardiovascular event was the earliest of stroke, PE or AMI, and MACE was the earliest of stroke, AMI or cardiovascular death defined in methods
a Models adjusted for age at index, sex, education, employment, pre-index comorbidities (cardiovascular disease, asthma, cancer, diabetes) and pre-index medication use (oral corticosteroids, inhaled corticosteroids, statins).
b Number of observations: overall, 97,186; moderate, 86,323; severe, 10,863.
c Number of observations: overall, 169,087; moderate, 145,984; severe, 23,103.

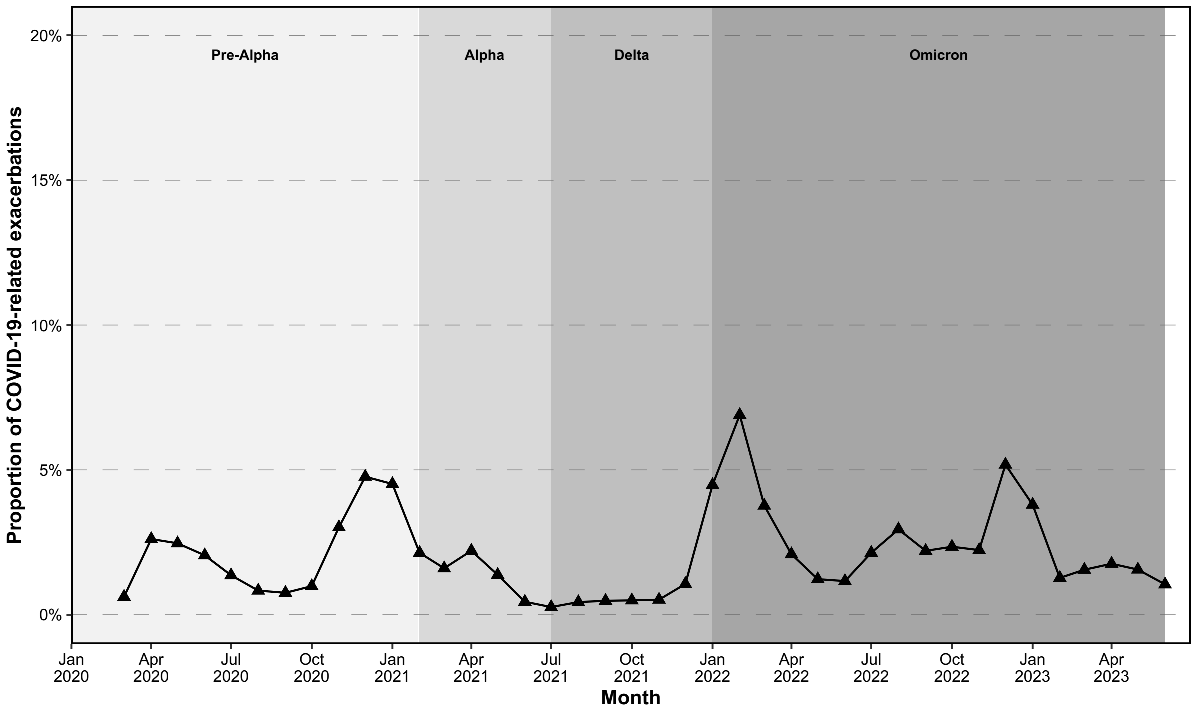

***Figure E1*. Monthly variation in the proportion of COVID-19-related exacerbations among COPD patients in Sweden from March 2020 to June 2023.**
